# Collision Tumors Involving Metastatic Carcinoma and Plasma Cell Myeloma: Report of Two Cases

**DOI:** 10.1155/crip/9279261

**Published:** 2026-04-25

**Authors:** Ahmed A. Ahmed, Muhammad Hassaan Khalid, Asad Ur Rehman, Nghia D. Nguyen, Xinhai Robert Zhang

**Affiliations:** ^1^ Department of Pathology, Microbiology and Immunology, University of Nebraska Medical Center, Omaha, Nebraska, USA, unmc.edu; ^2^ Department of Pathology and Laboratory Medicine, The University of Texas Health Science Center at Houston, McGovern Medical School, Houston, Texas, USA, uth.edu

**Keywords:** carcinoma, collision tumors, metastatic, myeloma, synchronous

## Abstract

Synchronous coexistence of plasma cell neoplasms (PCNs) and metastatic carcinoma is not a common phenomenon and poses a significant diagnostic challenge requiring meticulous histopathological and immunophenotypic analysis for accurate lineage assignment. We report two cases of collision tumors comprising PCN and metastatic lung carcinoma. Case 1 involved a 68‐year‐old male with metastatic non–small cell lung adenocarcinoma who developed intracranial lesions; resection revealed metastatic adenocarcinoma intermingled with clonal plasma cells. Case 2 was a 73‐year‐old woman with a lytic iliac crest lesion whose bone marrow and lesion biopsies showed diffuse infiltration by both carcinoma and atypical plasma cells. Comprehensive immunohistochemical profiling, in situ hybridization (ISH), and molecular studies were performed. In both cases, histology demonstrated two distinct, intermingled cell populations without transitional features. IHC was critical for confirmation: the carcinoma cells expressed epithelial markers (AE1/AE3), whereas the plasma cell components were positive for CD138. ISH in Case 1 and flow cytometry in Case 2 confirmed clonality (kappa light‐chain restriction). The final diagnoses were collision tumors of metastatic non–small cell lung adenocarcinoma with synchronous PCN (Case 1) and metastatic small cell carcinoma with synchronous PCN (Case 2). There are sporadic reports of PCN coexistent in the same lesion with solid tumors. These cases reveal the critical role of the pathologist in diagnosing collision tumors. IHC panel, including cytokeratins, CD138, and MUM1, can help in distinguishing these dual populations and avoiding diagnostic pitfalls.

## 1. Case Report

Herein, we present two interesting cases with the diagnoses of synchronous plasma cell neoplasm (PCN) and metastatic carcinoma.

Case (1): A 68‐year‐old male with a known history of Stage IV non–small cell lung carcinoma (NSCLC) adenocarcinoma subtype presented with acute neurological deficits. On admission, laboratory evaluation demonstrated hypochromic normocytic anemia and mild leukocytosis with a white blood cell count of 12 × 10^3^/*μ*L (reference range: 3.9‐10 × 10^3^/*μ*L). Imaging studies revealed multiple intracranial and spine lesions without evidence of any pathological fractures. CT imaging demonstrated a right upper lobe (RUL) mass associated with extensive mediastinal, hilar, and supraclavicular lymphadenopathy, and widespread metastases involving the liver, peritoneum, bone (notably L3 vertebra), and brain. The patient subsequently underwent surgical resection of the brain lesions following multiple sessions of brain radiation therapy and sessions for spinal metastases. He was also actively receiving immunotherapy.

**Figure 1:**
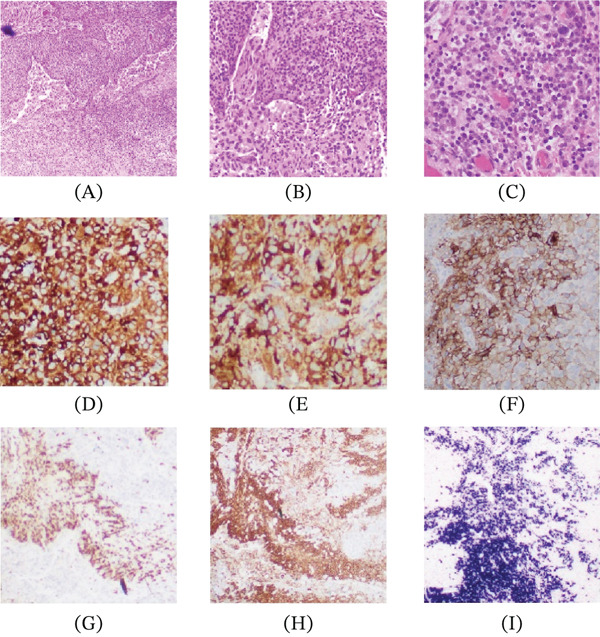
(A–C) H&E sections showing glandular structures of metastatic carcinoma infiltrating neural tissue and plasma cells (40×, 200×), (D) AE1/AE3, (E) Napsin A, (F) PDL‐1, (G) CD79a, (H) CD138, and (I) in situ hybridization (ISH) for kappa light chain.

Histologic evaluation of the resected brain lesions revealed two distinct populations: nests of metastatic carcinoma characterized by round glandular structures infiltrating neural tissue and interstitial clusters and nodules of mature‐appearing plasma cells (Figure 1A–C). Immunohistochemical (IHC) analysis showed that the carcinoma cells were positive for AE1/AE3, CK7, TTF‐1, Napsin A, PD‐L1, and ROS (Figure 1D–F) and were negative for P40, Synaptophysin, and ALK. These findings were consistent with metastatic lung adenocarcinoma. On the other hand, the plasma cells were positive for CD79a and CD138. In situ hybridization (ISH) showed kappa restriction of the plasma cells (Figure 1G–I). Molecular testing revealed PD‐L1 100% (TPS ≥ 50%) and a KRAS Q61R mutation, with negative results for EGFR, BRAF, and ERBB2/HER2. The findings suggested the presence of a collision tumor consisting of metastatic NSCLC and synchronous PCN with kappa light‐chain restriction.

The patient underwent additional Gamma Knife radiosurgeries for progressive intracranial metastases. Despite ongoing immunotherapy and localized radiotherapy, he exhibited continued systemic progression and functional decline.

Case (2): A 73‐year‐old female with a history of spinal tumor with lytic bone metastases presented with multiorgan failure, including respiratory and renal dysfunction, hypercalcemia, and sepsis. On admission, laboratory evaluation demonstrated hypochromic normocytic anemia and mild leukocytosis of 11.83 × 10^3^/mm^3^ (reference range: 3.9–10 × 10^3^/mm^3^). Renal function tests showed elevated serum creatinine at 2.81 mg/dL (reference range: 0.7–1.3 mg/dL). Serum Protein Electrophoresis (SPEP) revealed monoclonal gammopathy (MGUS) with an elevated kappa/lambda ratio. Imaging studies revealed diffuse skeletal and visceral metastases, with a prominent lytic lesion in the left iliac bone measuring up to 6 cm and pathological spinal fractures. Interventional radiology performed a bone marrow biopsy and fine needle aspiration of the left iliac crest for further evaluation.

**Figure 2:**
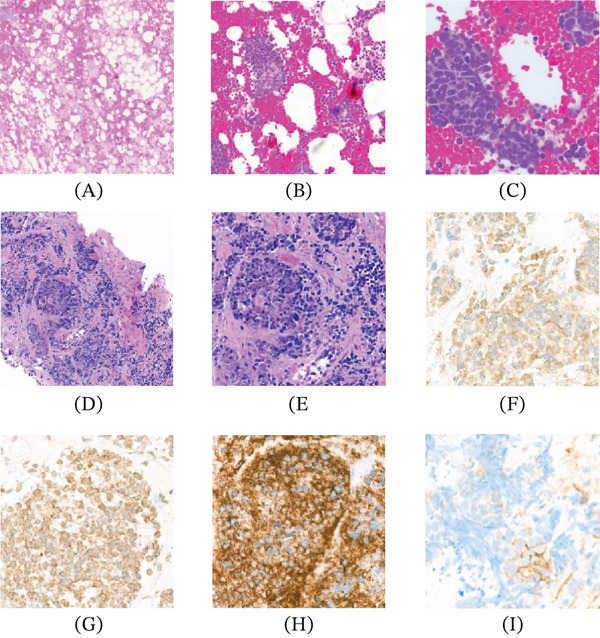
(A–C) H&E sections showing bone marrow involved by atypical plasma cells (40×, 200×), (D–E) H&E sections showing left iliac crest infiltration by carcinoma cells admixed with plasma cells, (F) Synaptophysin, (G) Chromogranin, (H) CD56, and (I) CD138.

Histologic evaluation of the bone marrow biopsy revealed a 10% interstitial infiltrate of mature, oval plasma cells. The bone marrow exhibited sheets of atypical plasma cells within a hypocellular bone marrow (Figure 2A–C). The atypical plasma cells were positive for CD79a, MUM‐1, and CD138 (20%). Flow cytometry identified plasma cells with cytoplasmic kappa light‐chain restriction. The malignant plasma cells were positive for CD138, CD56, and CD117 and were negative for CD19, CD20, CD38, and CD45. Cytogenetic analysis revealed complex chromosomal aberrations. The second biopsy of the lytic lesion from the left iliac crest demonstrated diffuse infiltration by clusters of carcinoma cells admixed with plasma cells (Figure 2D–E). IHC analysis showed that the carcinoma cells were positive for AE1/AE3, Synaptophysin, Chromogranin, and CD56 (Figure 2F–H) whereas negative for TTF1 and Napsin A. CD138 showed focal positivity (Figure 2I). Kappa and lambda stains were inconclusive due to background staining. Morphologically, the carcinoma cells displayed a more cohesive arrangement compared to the malignant plasma cells. The presence of both neoplasms within the same lesion confirmed the diagnosis of synchronous metastatic small cell carcinoma and PCN. The patient′s clinical status deteriorated rapidly, and she expired within days of the diagnosis.

## 2. Discussion

Collision tumors represent uncommon pathological entities characterized by the coexistence of two histogenetically distinct neoplasms within the same anatomical site, maintaining independent morphologic, immunophenotypic, and molecular profiles without evidence of transitional features [1]. Recognition relies on identifying dual neoplastic cell populations on H&E sections and confirming lineage through IHC and molecular tests to exclude clonal overlap. Collision tumors signal complex disease biology and typically indicate poor prognosis, as treatment must target both components while considering drug interactions and systemic disease extent [2].

The synchronous presentation of PCN, such as multiple myeloma or plasmacytoma, with a solid neoplasm, particularly NSCLC or renal cell carcinoma (RCC), is not a common event. While some case reports have documented this association, the occurrence of both neoplasms within the same bone lesion, forming a true collision tumor, represents a remarkable phenomenon [3–5]. The difficulty in such situations rises from the fact that osteolytic bone lesions are a common feature of both multiple myeloma and metastatic carcinoma. Although certain radiographic features, such as the well‐defined “punched out” lesions typical of myeloma or the pedicle involvement often seen in metastatic disease, may provide clues, considerable overlap precludes a definitive diagnosis [6].

By morphology, carcinoma cells are typically large with nuclear atypia and arranged in cohesive nests, glands, or islands, accompanied by a desmoplastic stroma. In contrast, neoplastic plasma cells are relatively small, with plasmacytoid morphology (eccentric clock‐face chromatin and perinuclear hof), and form generally disperse, sheet‐like, or interstitial patterns. IHC is important for lineage confirmation. Carcinomas are characterized by expression of cytokeratins (e.g., AE1/AE3, CK7, CK20) and, in some cases, organ‐specific transcription factors that aid in identifying the primary site. PCNs are confirmed by CD138 and MUM1/IRF4 positivity. MUM1/IRF4, a nuclear marker with high specificity for normal and neoplastic plasma cells, is consistently negative in epithelial malignancies and helps in the differential diagnosis [7]. Interestingly, CD138 can be aberrantly expressed in some epithelial malignancies, including those of prostate, colon, kidney, and liver origin [8].

The pathogenesis underlying collision tumors remains incompletely understood and is likely multifactorial. Proposed hypotheses include (1) coincidental occurrence of two unrelated neoplasms; (2) therapy‐related oncogenesis, wherein cytotoxic or radiotherapeutic interventions for one malignancy induce a second; (3) the “seed and soil” hypothesis, positing that the microenvironment created by one tumor, via elaboration of cytokines and growth factors such as IL‐6, a factor implicated in both RCC and PCN pathogenesis, fosters the development of the other; (4) bidirectional cellular crosstalk with consequent perturbation of local immune surveillance; and (5) underlying genetic predispositions that increase susceptibility to multiple primary neoplasms [9–11]. Reactive bone marrow plasmacytosis in the setting of a carcinoma may serve as a precursor lesion for subsequent PCN, whereas immune dysregulation inherent to PCN, mediated by CD38 overexpression or impaired T‐cell function, could compromise antitumor immunity and facilitate emergence of a secondary solid malignancy [12, 13].

Although the exact molecular drivers of NSCLC remain unclear, epidermal growth factor receptor (EGFR) mutations are among the most frequently implicated genetic alterations [14]. Notably, EGFR mutations have also been detected in PCN cells, and emerging evidence suggests that PCN may acquire resistance to EGFR inhibitors via activation of the pentose phosphate pathway [15, 16]. Tumor necrosis factor‐alpha (TNF‐*α*) exhibits a dual role in PCN pathophysiology, contributing to both B‐cell proliferation and apoptosis in neoplastic plasma cells [17]. Furthermore, elevated CD38 expression has been associated with immune dysfunction and may contribute to pathogenesis in both PCN and lung cancer [18, 19].

Collision tumors often portend a more complex disease biology and potentially poorer prognosis. Treatment must be carefully adjusted to address both components, considering efficacy, timing, and potential interactions among modalities including combination chemotherapy, proteasome inhibitors, immunomodulatory drugs, radiation, and surgery. The use of agents with potential activity against both tumor types, such as alkylating agents or CD38‐targeted therapies, may offer strategic advantage.

The synchronous coexistence of metastatic carcinoma and PCNs represents a challenging scenario. Clinical differentiation can be challenging, due to overlapping features such as pain, cytopenias, and end‐organ failure. Accurate diagnosis requires adequate sampling with histopathologic evaluation. Further investigation into the molecular drivers and microenvironmental interactions underlying these rare coexisting malignancies may help form more accurate diagnostic algorithms.

## Author Contributions

A. A.: contributed to the conception, design, data interpretation, and manuscript drafting. M. H. K. and A. U. R.: assisted in data collection, histopathologic review, and literature analysis. A. N.: contributed to clinical data acquisition and reviewed the manuscript. X. R. Z.: supervised the project, provided critical revision of the manuscript, and approved the final version for submission.

## Funding

No funding was received for this manuscript.

## Ethics Statement

According to institutional policy, ethical approval was not required for case reports.

## Consent

This study has obtained IRB approval from the University of Texas Health Science Center at Houston, and the need for informed consent was waived. For this type of study, consent for publication is not required.

## Conflicts of Interest

The authors declare no conflicts of interest.

## Data Availability

The data that support the findings of this study are available on request from the corresponding author. The data are not publicly available due to privacy or ethical restrictions.
